# Editorial: Nutraceuticals in sports

**DOI:** 10.3389/fnut.2026.1995867

**Published:** 2026-09-28

**Authors:** Francisco J. Osuna-Prieto, Paulina Mazur-Kurach, Florentyna Tyrała, Aleksandra Bojarczuk

**Affiliations:** 1 Hospital Universitari Joan XXIII de Tarragona, Institut de Recerca Biomèdica Catalunya Sud (IRB CatSud), Tarragona, Spain; 2 Centro de Investigación Biomédica en Red de Diabetes y Enfermedades Metabólicas Asociadas (CIBERDEM) - Instituto de Salud Carlos III (ISCIII), Madrid, Spain; 3 Department of Sports Medicine and Human Nutrition, Institute of Biomedical Sciences, University of Physical Culture in Krakow, Krakow, Poland; 4 Department of Sports Dietetics, Faculty of Physical Education, Gdansk University of Physical Education and Sport, Gdansk, Poland; 5 Department of Molecular Biology, Faculty of Physical Education, Gdansk University of Physical Education and Sport, Gdansk, Poland

**Keywords:** dietary supplements, exercise performance, nutraceuticals, recovery, sports nutrition, training

The growing interest in nutraceuticals in sports science reflects increasing attention to bioactive compounds that influence physiological processes relevant to performance, recovery, and adaptation. This Research Topic brings together mechanistic studies, human interventions, observational research, and evidence syntheses that collectively illustrate both the promise and the complexity of nutraceutical applications in exercise and sport. A recurring theme throughout the Research Topic is that biologically plausible mechanisms do not always translate into consistent performance benefits, underscoring the need for evidence-informed, context-specific implementation.

A central theme emerging across the Research Topic is the modulation of mitochondrial function, oxidative stress, and exercise-induced fatigue. Several studies converged on the concept that nutraceutical interventions may influence endurance performance not necessarily through direct energetic enhancement, but potentially by modulating redox balance and attenuating excessive physiological stress. In a preclinical study, Mizuno et al. found that hydrogen-rich water increased running distance in male mice after 4 and 6 weeks, but not after 1 or 2 weeks. After 4 weeks of consumption, it also reduced post-exercise markers of oxidative stress and inflammation and serum lactate dehydrogenase activity, whereas creatine kinase activity did not differ between groups. Because these effects occurred without upregulation of nuclear factor erythroid 2-related factor 2 (Nrf2)-related antioxidant defenses, the authors proposed direct reactive oxygen species (ROS) scavenging or suppression of ROS production as possible mechanisms, although neither was directly assessed. Wang et al., in a systematic review and meta-analysis of 26 randomized controlled trials (RCTs) involving 668 healthy participants, reported improvements in maximal oxygen uptake (VO_2_max), time to exhaustion, and time-trial performance following *Rhodiola rosea* supplementation, together with favorable changes in markers of antioxidant capacity, muscle damage, and lactate. However, heterogeneity was moderate to high for several biomarkers, and differences in study and intervention protocols, the limited number of studies, and small sample sizes warrant cautious interpretation. Likewise, the systematic review and meta-analysis by Shu et al., which quantitatively synthesized eight of 14 RCTs (288 of 528 athletes), identified product- and outcome-specific effects of fungal supplementation in adult athletes. *Cordyceps sinensis* improved selected endurance-related outcomes, including ventilatory threshold and peak oxygen uptake (VO_2_peak), whereas the significant findings for *Ganoderma lucidum* concerned biochemical, antioxidant, and hematological markers. However, the limited evidence base, variation in supplement formulations, and methodological concerns limit the strength and generalizability of the evidence. Taken together, these findings indicate that changes in oxidative stress biomarkers should not be interpreted in isolation but alongside performance outcomes, intervention duration, training context, and recovery status. The complex relationship between biomarker changes and functional effects is also evident in emerging interventions such as urolithin A, a compound associated with mitochondrial quality-control pathways. In a single-blind pilot randomized study, Monsalve Acevedo et al. reported greater improvements in Yo-Yo intermittent recovery performance and countermovement jump height with urolithin A than with placebo in 20 male academy soccer players. No significant group-by-time interactions were observed for antioxidant outcomes, precluding conclusions that urolithin A preserved antioxidant status relative to placebo. Although the performance findings provide preliminary evidence in an athletic population, broad confidence intervals, baseline imbalance, and between-group differences in training exposure and adherence warrant caution. Mitochondrial function and mitophagy were not assessed, leaving the proposed mechanisms speculative.

Another major theme emerging across the Research Topic is the growing recognition that nutraceuticals may influence cognitive performance, psychophysiological resilience, and athletes' wellbeing beyond purely physical outcomes. Feng et al. found that acute caffeine ingestion improved anticipation accuracy and reaction time under fatigue, supported repeated-sprint fatigue resistance, and was associated with better overall agility in 15 elite male badminton athletes. Interestingly, caffeine did not improve fine technical skills such as smash accuracy, emphasizing that enhanced physiological arousal or cognitive responsiveness does not necessarily translate into improvements across all sport-specific domains. This distinction is highly relevant for precision performance nutrition, where interventions may differentially influence endurance, cognition, motor control, or technical execution depending on task demands.

Beyond experimentally assessed performance effects, Karam et al. found that 38% of 80 surveyed elite Canadian athletes (51 males and 29 females) had used cannabidiol (CBD), although only nine reported current use. Users perceived benefits for sleep, relaxation, training-related pain, and recovery, but these were subjective. Frequent reliance on friends and the internet as initial information sources, along with concerns about supplement contamination and inadvertent anti-doping rule violations, underscores the need for evidence-based guidance.

The expansion of sports nutrition into non-traditional athletic populations further broadens the field. Mazur-Kurach et al. reported generally low diet quality and abnormalities in selected health markers among 174 Polish professional and semi-professional male e-sports players. However, associations between diet quality and metabolic indicators were weak, and multidimensional analyses did not identify diet quality as a significant predictor of the assessed health indicators, precluding causal conclusions.

Nutraceutical efficacy also appears highly context-, dose-, and population-dependent. In 171 recreationally active male university students, Wang and Wu identified a U-shaped dose-response relationship for carbohydrate-protein co-ingestion during rowing, with the lowest dose producing a greater rowing distance than several higher doses. Similarly, Kong et al. reported time-dependent and inconsistent effects of acute *Tribulus terrestris* supplementation following blood-flow-restriction priming in 20 elite male basketball players. Compared with placebo, supplementation increased the peak rate of force development immediately after priming, and the modified reactive strength index at 4 and 16 min, but jump height and power were lower at later time points. No significant between-condition differences were observed for maximum combined or relative force. Null or limited effects were also reported. Durkalec-Michalski et al. found that high-dose individualized β-hydroxy-β-methylbutyrate (HMB) supplementation did not alter body composition among 90 trained and untrained men, either during 3 weeks under usual conditions or during a subsequent 3 weeks combined with additional high-intensity functional exercise. Likewise, a meta-analysis of 23 RCTs involving 1,146 participants (897 men and 249 women) by Xiao et al. found that protein supplementation during endurance training improved time to exhaustion but not time-trial performance, sprint performance, VO_2_max, peak power, body weight, or body fat. Lean body mass did not increase significantly, and no significant moderators of VO_2_max or lean body mass were identified.

These findings complement the network meta-analysis of 19 RCTs involving 997 older adults by Ma et al., in which protein supplementation combined with resistance training was most effective for improving muscle strength in healthy older adults, whereas creatine produced the greatest gains in muscle mass. HMB did not significantly benefit either outcome. Together, these studies show that nutraceutical effects depend on dose, population, and the specific physiological or performance outcome targeted. Across the Research Topic, small sample sizes, short interventions, inconsistent dosing, and heterogeneous endpoints limit interpretation, while findings from animal models cannot be directly extrapolated to athletic performance. Future studies should use adequately powered longitudinal designs integrating mechanistic and functional outcomes, include more female and elite athletes, and assess interventions in ecologically valid training environments. Multi-omics and individualized biomarker approaches may help identify responder phenotypes, but isolated biomarker changes should be interpreted alongside meaningful functional and performance outcomes.

Overall, the evidence indicates that nutraceutical efficacy depends on the compound, dose, population, training context, and target outcome. Integrating evidence from mechanistic experiments, human interventions, observational studies, and evidence syntheses supports targeted rather than universal supplementation strategies aligned with clearly defined physiological and performance objectives.

